# Recent Advances in Diagnosis and Therapy of Angioimmunoblastic T Cell Lymphoma

**DOI:** 10.3390/curroncol28060456

**Published:** 2021-12-20

**Authors:** Mostafa F. Mohammed Saleh, Ahmed Kotb, Ghada E. M. Abdallah, Ibrahim N. Muhsen, Riad El Fakih, Mahmoud Aljurf

**Affiliations:** 1 Adult Hematology, Transplantation and Cellular Therapy Section, Oncology Center, King Faisal Specialist Hospital and Research Center, Riyadh 11211, Saudi Arabia; aahmedkotb@kfshrc.edu.sa (A.K.); relfakih1@kfshrc.edu.sa (R.E.F.); maljurf@kfshrc.edu.sa (M.A.); 2 Clinical Hematology Unit, Department of Internal Medicine, Faculty of Medicine, Assiut University, Assiut 71515, Egypt; ghadaelsayed2008@yahoo.com; 3 Clinical Hematology Unit, Department of Internal Medicine, Faculty of Medicine, Zagazig University, Zagazig 44519, Egypt; 4 Department of Medicine, Houston Methodist Hospital, Houston, TX 77030, USA; inmuhsen@houstonmethodist.org

**Keywords:** angioimmunoblastic T cell lymphoma, diagnosis, therapy

## Abstract

Angioimmunoblastic T cell lymphoma (AITL) is a common subtype of mature peripheral T cell lymphoma (PTCL). As per the 2016 World Health Organization classification, AITL is now considered as a subtype of nodal T cell lymphoma with follicular helper T cells. The diagnosis is challenging and requires a constellation of clinical, laboratory and histopathological findings. Significant progress in the molecular pathophysiology of AITL has been achieved in the past two decades. Characteristic genomic features have been recognized that could provide a potential platform for better diagnosis and future prognostic models. Frontline therapy for AITL was mainly depending on chemotherapy and the management of relapsed or refractory AITL is still unsatisfactory with a very poor prognosis. Upfront transplantation offers better survival. Novel agents have been introduced recently with promising outcomes. Several clinical trials of combinations using novel agents are underway. Herein, we briefly review recent advances in AITL diagnosis and the evolving treatment landscape.

## 1. Introduction

Angioimmunoblastic T cell lymphoma (AITL) is a subtype of peripheral T cell lymphoma (PTCL) accounting for 15–30% of the cases with unique clinicopathologic and genetic features. Previously, it was considered as an immune reactive process with a number of descriptive names were used in the past (immunoblastic lymphadenopathy, angioimmunoblastic lymphadenopathy with dysproteinemia, lymphogranulomatosis X). Identification of clonal T cells with the advent of immunophenotyping had led to the recognition of AITL as a malignant entity in the T cell lymphoma classification [1,2]. 

The revision of the WHO classification divided the nodal T cell lymphoma with follicular helper T (TFH)-cell phenotype, into three subgroups: AITL, follicular T cell lymphoma, and nodal peripheral T cell lymphoma with TFH phenotype with the last 2 being new provisional entities [2].

The pathogenesis of AITL is not fully understood. A number of reports showed that AITL may be linked to viral infections, such as Epstein-Barr virus (EBV). High EBV viremia upon presentation was associated in some reports with a worse response, disease progression, or evolution to aggressive B cell lymphoma [3,4,5,6,7]. Neoplastic TFH cells are postulated to have a role in AITL initiation and development through the deregulation of cytokines, such as interleukin-21 (IL-21), IL-4, and CXCL13 secreted by TFH cells that play important roles in germinal center cells’ interactions and activation [8]. In addition to the recent advances in its phenotypic characterization, the genetics and molecular mechanisms underlying AITL are being recently unfolded indicating dysregulation of several biologic pathways involved in lymphomagenesis [9]. 

## 2. Epidemiology

AITL is the second common subtype of PTCL with variable geographical incidence (16% in the USA, 18% in Asia and 29% in Europe) but only accounts for approximately 1% to 2% of all non-Hodgkin’s lymphoma (NHL) [2,10].

A large population-based study using the Surveillance, Epidemiology, and End Results (SEER) database reported 1207 AITL patients, the median age at diagnosis was 69 years. The incidence was slightly higher in males (51.5%) and advanced-stage (III to IV) disease was reported in 80% of patients [11]. 

## 3. Clinical Presentation and Diagnosis

Clinically, AITL-patients are typically symptomatic upon presentation. Systemic symptoms are commonly reported, in addition to generalized lymphadenopathy, including B-symptoms, skin rash, pleural effusions, arthritis, symptoms related to polyclonal hypergammaglobulinemia, and autoimmune phenomena, such as hemolytic anemia or immune thrombocytopenia [12,13].

The workup starts with routine testing, such as complete blood count (CBC), complete metabolic panel, lactate dehydrogenase (LDH) level, testing for EBV, hepatitis B and C, HIV and human T-lymphotropic virus. Laboratory findings in AITL include cytopenias, elevated inflammatory markers, LDH, positive autoimmune phenomena (positive coombs’ test, thyroid dysfunction, etc.) and polyclonal hypergammaglobulinemia. Bone marrow biopsy and PET/computed tomography imaging are needed for staging.

Pathological confirmation on a biopsy is the cornerstone to making a diagnosis. The lymph node pathology usually shows partial or total effacement of the lymph node architecture. The infiltrate is usually diffuse or paracortical, composed of a polymorphous population of atypical T cells, usually small to medium in size with clear cytoplasm, clustering around high endothelial venules and enwrapped by follicular dendritic cell (FDC) meshworks. There are scattered immunoblasts, histiocytes, plasma cells, and eosinophils with prominent networks of arborizing high endothelial venules [14]. Three overlapping histologic patterns have been identified in AITL. Pattern I has limited nodal involvement and hyperplastic follicles while patterns II and III, in the majority of AITL cases, show typical AITL features described before with or without regressed follicles. Histologic progression from pattern I to typical AITL has been reported in 23% of cases, it has been recognized as the development of secondary B-cell lymphoma (often EBV-associated), rather than the progression of T cell neoplasm [15]. Immunohistochemical (IHC) examination is critical to establish AITL diagnosis, neoplastic cells are typically positive for T cell markers as CD4, CD5, and CD2, as well as markers of TFH cells, such as CD10, CXCL13, ICOS, BCL6, and PD1 [16,17,18]. Immunoblastic cells are large with basophilic cytoplasm and polytypic, CD20 and Epstein Barr Virus (EBV) –encoded RNA positive. Follicular dendritic cells could be recognized outside of the follicles and often around vessels with positive stains for FDC, such as CD21 and CD23 [12,19]. The 2016 WHO classification recognized that at least two (ideally three) TFH markers have to be expressed by the atypical cells to diagnose a case of PTCL with TFH phenotype [2].

CD30 expression is a common finding in AITL. Of 51 cases (43 AITLs and eight PTCL-NOSs) most (90%) had CD30 expression by IHC (range; 1% to 95%), with high levels (>50%) in 14% of cases [20]. Skin involvement is less likely to have typical AITL features seen in lymph nodes, IHC markers of TFH could be helpful but not distinctive. Flow cytometry of bone marrow samples shows a variable phenotype of sCD3(–/dim)/CD4+/CD10 for neoplastic T cells involved [14].

## 4. Genomics of AITL

Genomics of AITL is rapidly evolving. Conventional karyotyping showed clonal abnormalities defining a stemline, with one or more sidelines in approximately 30–50% of the cases. The most common abnormalities for AITL include gain of chromosomes 5q (55%), 21 (41%) and 3q (36%), concurrent trisomies of 5 and 21 (41%), and loss of 6q (23%) [21,22].

Over the past two decades, recent advances in next-generation sequencing identified characteristic recurrent molecular mutations in AITL. This allowed a better understanding of different biologic pathways involved in AITL pathogenesis Recurrent genetic abnormalities in ras homolog family member A (*RHOA*) (50–70%) and in genes of the epigenetic modulators, tet methyl cytosine dioxygenase 2 (*TET2*) (47–83%), DNA methyltransferase 3 alpha (*DNMT3A*) (20–30%), isocitrate dehydrogenase 2 (*IDH2*) (20–45%) have been described [23,24]. 

Wellmesen and colleagues analyzed the association between *RHOA* mutational status and other recurrent mutations using the data from large sequencing studies and proposed three potentially AITL lymphomagenic pathways. The classic one with concomitant *RHOA G17V* mutation and mutations in *TET2*, *DNMT3A*, *IDH2* and *CD28*. Another alternative pathway with mutations in *VAV1* or potentially yet unidentified mutations in members of the Rho family of GTPases or their regulatory proteins. Third, AITL cases with unknown mutations could be related to genetic alteration in pathways regulating TFH differentiation. They assumed those pathways could be reflected in different clinical behavior seen in AITL patients [25].

An interesting theory of multistep tumorigenesis has been suggested, in which initial *TET2* and *DNMT3A* mutations occur in premalignant cells followed by subsequent mutations in genes of T cell function (*RHOA*, *VAV1*, *PLCG1*, *CD28*, and others) leads to AITL [26]. Another exciting reflection related to *DNMT3A* and *TET2* mutations in AITL exists. Both are also common in clonal hematopoiesis (CH) and myeloid neoplasms (MNs). AITL patients showed a higher incidence of CH and MNs signifying that they arise from the divergent evolution of a common CH clone [27].

T cell receptor (TCR) signaling has been postulated to be involved in the pathogenesis of TFH-derived PTCLs. TCR signaling genes are actively and exclusively mutated in half of the TFH-derived lymphomas. The five commonly mutated genes (*PLCG1, CD28, PIK3* elements, *GTF2I*, *CTNNB1)* were reported in 14% to 5% of patients [28]. 

In another study, clonal gene rearrangements were found in the majority of patients (87%). The immunoglobulin gene (IG) rearrangements were noted in 55 % and the TCR gene in 58% while concurrent IG and TCR gene rearrangements were observed in 14 cases (25%). Fifteen cases of reactive lymphoid hyperplasia were analyzed in this study as well and there were no IG or TCR gene rearrangements in these cases [29]. 

*IDH2^R172^* mutated AITL showed a specific gene expression signature with downregulation of T_H1_ differentiation related genes (e.g., *STAT1* and *IFNG*) and a prominent enhancement of an interleukin 12-induced gene signature. These cases are also characterized by hypermutation of genes involved in TCR signaling and T cell differentiation which can contribute to lymphomagenesis [30].

Acquisition of B-cell-specific mutations represented by *NOTCH1* and others has been reported significantly in AITL and this may account for the frequent occurrence of monoclonal expansion of B cells and development of B-cell lymphomas noted in AITL [31].

Sequencing discoveries of molecular mutations implicated in AITL are rapidly evolving. The challenge will be how to make use of the best of it for diagnostic and prognostic purposes to set up a standardized genomic panel and develop novel risk assessment models.

## 5. Prognosis 

Till 2010, no major progress has been made in AITL. No survival differences were noted among 1207 patients with AITL subgroups diagnosed in five periods (1992 to 1998, 1999 to 2001, 2002 to 2004, 2005 to 2007, and 2008 to 2010). This analysis was done using the SEER database (1973–2010) to determine the prognostic factors and survival trends of AITL. Adverse predictors for OS and disease-specific survival were age older than 70 years, advanced-stage disease and male sex [11]. 

Different prognostic factors and models have been proposed for outcome-prediction in PTCL including AITL patients, listed in Table 1. As per the International Peripheral T cell Lymphoma Project, among 1314 patients, 243 (18.5%) were diagnosed with AITL [12]. For better risk assessment of AITL patients, prognostic models were evaluated at presentation, including the standard International Prognostic Index, the Prognostic Index for Peripheral T cell Lymphoma and the alternative Prognostic Index for AITL (PIAI), comprising: age > 60 years, PS ≥ 2, ENSs > one, B symptoms, and platelet count <150 × 10^9^/L. The PIAI, compared to the others, showed more predictive value of low- and high-risk subgroups of patients with AITL with a 5-year survival of 44%, and 24%, that significance was maintained in validation analysis of the GELA study cohort [12].

A novel prognostic score (AITL score) combining age, C-reactive protein, Eastern Cooperative Oncology Group performance status, and β2 microglobulin has been recently reported. It stratified AITL patients into low-, intermediate-, and high-risk subgroups with 5-year OS rates of 63%, 54%, and 21%, respectively, with better discernment, compared to established prognostic indices [32]. 

A recent comparison of prognostic scores among transplant-ineligible patients with PTCL-NOS and AITL demonstrated the international prognostic index (IPI) had better c-statistics (>0.7) for OS in PTCL-NOS compared to the prognostic index for T cell lymphoma (PIT), modified PIT, and the International Peripheral T Cell Lymphoma Project for overall survival. IPI was exclusively impactful for investigating the risk factors associated with outcomes AITL [33]. 

In a retrospective analysis of 207 Japanese patients with AITL, The International Prognostic Index (IPI) and the prognostic index for PTCL NOS were predictive for OS. Multivariate analysis recognized age older than 60 years, elevated white blood cell (WBC) and IgA levels, the presence of anemia and thrombocytopenia, and extranodal involvement at >1 site as significant prognostic factors for OS. IgA, anemia, and mediastinal lymphadenopathy were significant prognostic factors for PFS [34].

Other factors based on infectious origin, immune-histochemical features and genomics have been shown to impact AITL outcome. Among younger patients (≤60 years) with AITL, an EBER^+^ status had a significantly better prognosis compared to an EBER^−^ status. A new prognostic model, based on three adverse factors EBER negative status, thrombocytopenia and elevated serum IgA level classified the patients into two risk groups: low risk (no or 1 adverse factor) and high risk (two or three adverse factors). This new model showed that both OS and PFS were significantly linked to the level of risk [35].

Peripheral Epstein-Barr viral load at diagnosis (>100 copy/μg DNA) was related to shorter PFS [36]. *TET2* mutations and *CD28* mutations showed to be associated with worse PFS and OS, respectively, whereas mutations in *IDH2*, *RHOA*, and genes related to the TCR pathway had no impact on survival [37].

A genomic based prognostic model including B-cell, monocytic and p53-induced genetic signatures has been significantly correlated with AITL outcomes. B-cell-associated signature showed better outcome, while the other two were associated with worse survival [38].

The predictive value of the PET/CT has a great significance in the management of lymphoma. In AITL, baseline PET/CT with high maximum standardized uptake value (SUVmax) and high total metabolic tumor volume (TMTV) showed poor outcomes. Interim PET/CT with Deauville score ≥3 and percent decrease of SUV max less than 60% are associated with poor PFS and OS [39,40,41].

**Table 1:** Prognostic models and factors of AITL.

Model	Factors	Score	Impact OS/PFS %
International Prognostic Index (IPI) [12]	-Age ≥ 60 years -Stages III to IV disease -Lactic dehydrogenase (LDH) > normal -Extranodal sites (ENSs) > one -Performance status (PS) ≥ 2	0–1 2 3 4–5	@5 years 56/34 @5 years 38/21 @5 years 20/12 @5 years 25/16
Prognostic Index for Peripheral T cell Lymphoma [12]	-Age ≥ 60 years -PS ≥ 2 -LDH > normal -Bone marrow involvement	0–1 2 3–4	@5 years 46/22 @5 years 19/12 @5 years 30/22
Prognostic Index for AITL (PIAI) [12]	-Age > 60 years, -PS ≥ 2, -ENSs > one, -B symptoms, and -Platelet count < 150 × 10^9^/L	Low-risk group (0–1 factors) High-risk group (2–5 factors)	@5 years 44/28 @5 years 24/15
Eladl et al., 2020 [35]	-EBER negative status, -Thrombocytopenia -Elevated serum IgA level	Low risk (0–1 factor) High risk (2–3 factors)	@3 years 91/49 @3 years 18/0
AITL score [32]	-Age -PS -C-reactive protein -β2 microglobulin	Low risk Intermediate High	@5 years 63/- @5 years 54/- @5 years 21/-
Iqbal et al. (Gene expression model) [38]	-B cell (GCB cell signature) -Monocytic/dendritic signature -TP53-induced gene signature	Good prognosis Poor prognosis Poor prognosis	@5 years 56–64 @5 years 13–14 @5 years 13–14

Abbreviations: AITL, angioimmunoblastic T cell lymphoma; PFS, progression free survival; OS, overall survival; GCB cell, germinal center B cell; @, At.

## 6. Treatment

### 6.1. Frontline Therapy

The treatment landscape of AITL mirrors that of other nodal PTCLs. Typically, the frontline therapy is CHOP-like (cyclophosphamide, doxorubicin, vincristine, and prednisone), anthracycline based ± etoposide. The overall response (ORR) rate with CHOP-like regimens in PTCLs ranges between 70% and 79%, with a complete response (CR) rate around 39% [42]. The 5 years failure-free survival rate for patients with AITL treated with CHOP alone ranges from 13 to 20% [10,42,43]. The addition of etoposide to CHOP improved the progression free survival (PFS) when compared to CHOP alone in young and fit patients, with high toxicity noted in patients older than 60 [44,45]. Other studies showed that CHOP intensification caused prolonged cytopenias without clear benefit in PFS [46,47].

Adding different novel agents to the CHOP backbone has been tried to improve the CHOP efficacy in the front-line setting [48,49]. Table 2 summarizes the studies using CHOP like backbone therapy with the addition of other agents.

Biologic monoclonal antibodies directed against antigens of lymphoma cells proved striking improvement in B cell lymphoma. Similarly, the ECHELON-2, a phase III study, compared the efficacy and safety of Brentuximab Vedotin (BV) with CHP (without vincristine) to CHOP for CD30-positive PTCL. Higher ORR was noted in 83%. A statistically significant improvement in PFS (48.2 months) and OS (not reached) was achieved for patients treated on the BV combination arm. Febrile neutropenia and peripheral neuropathy were similar in both groups [50]. Adding rituximab (anti-CD20) targeting the intratumoral B lymphocytes was explored. The reported ORR was 80%, but the PFS was similar to CHOP alone [36]. 

Another monoclonal antibody, alemtuzumab (anti-CD52) was combined with CHOP in several trials. The reported response rates were possibly better than CHOP alone, but patients receiving alemtuzumab developed significant opportunistic infections [51,52,53,54,55,56,57]. 

PTCL, especially in the AITL subtype has an activated pathway of the transcription factor NF-κB. Bortezomib, a proteasome inhibitor with NF-κB inhibitory activity, was added to CHOP in a phase II trial for patients with stage III/IV PTCL. The ORR and CR rates were 87% and 76% respectively. However, the 3-year OS and PFS were not promising [58]. 

AITL is frequently associated with autoimmune phenomena; immunomodulation could theoretically have good disease control. Lenalidomide added to CHOP showed modest activity when used in elderly patients suffering from AITL. The PFS and OS rates at 2 years were 42.3% and 60.1% respectively [59]. In an interesting case report with a conflicting lymphoma consisting of AITL and DLBCL, CR was achieved with six cycles of lenalidomide combined with R-miniCHOP regimen followed by lenalidomide maintenance with sustained remission [60]. Low dose recombinant interferon alfa-2a was used historically as a single agent with limited efficacy [61]. 

The anti–angiogenesis agent bevacizumab was combined with CHOP in the E2404 study. High ORR was reported but was short lived and toxicity was unacceptably high including febrile neutropenia, congestive heart failure and gastrointestinal hemorrhage/perforation. PFS was 44% at one year, with a median OS of 22 months after three years of follow-up [62]. 

Epigenetic modifiers mutations have been recognized as a hallmark of AITL, which represent attractive therapeutic targets in this disease. Histone deacetylase inhibitors and hypomethylating agents showed promising results in the R/R setting Moving those agents to the frontline therapy has been studied and recently reported with disappointing results. In a phase III randomized study, romidepsin combined with CHOP (Ro-CHOP) compared to CHOP in patients with previously untreated PTCL, demonstrated similar response rates (ORR 63% vs 60%; CR + CRu rates 41% vs 37%). No statistically significant improvement was noted in PFS (12 vs 10.2 months) and OS (51.8 vs 42.9 months) [63]. Chidamide is a selective inhibitor of HDAC1, 2, 3, and 10 and is administrated orally. In a prospective, multicenter, single arm, phase 2 study, 6 cycles of Chi-CHOEP regimen were given as first line in 113 PTCL patients (AITL 41). ORR was 60.2% (AITL 65.9%), CR 40.7% (AITL 41.5%). Median PFS was 10.7 (AITL 9.6 months). Patients with AITL showed significantly inferior outcomes compared with ALK-ALCL and PTCL-NOS patients [64].

Alternative induction chemotherapy regimens other than CHOP were tried as well. Gemcitabine, cisplatin, prednisone and thalidomide (GDPT) were compared to CHOP in newly diagnosed PTCL. The CR, OS and PFS rates were in favor of the GDPT group. However, looking specifically at the AITL group of patients within the GDPT-treated group, the OS of AITL was shorter than that of the other subgroups of PTCL within the GDPT group (*p* = 0.001) [65]. 

Hematopoietic stem cell transplantation (SCT) is usually reserved for R/R patients. However, few studies investigated the efficacy of this approach in the frontline setting. Of the NLG-T-01 study, 115 PTCL patients underwent HDT/ASCT, 5-year OS and PFS were 51% and 44% respectively [66]. In a multicenter study, autologous SCT (ASCT) in CR1 significantly improved OS and PFS for patients with AITL compared to other PTCL subtypes. Superior survival was noted in patients with advanced stage and high IPI scores. On multivariable analysis, ASCT remained independently associated with improved survival [67]. 

In another trial looking at 285 adults allo-HSCT for non-primary cutaneous PTCL, with almost the third of patients being AITL, 138 patients were transplanted in the frontline setting (93 were in CR and 45 had partial remission). For all cohorts, including those with second or more CR/PR, OS was 65% at 2 years and 59% at 4 years and the cumulative incidence of relapse was 18% after 1 year and 19% after 2 years. In multivariate analysis, low Karnofsky performance score (<80%), grade III-IV acute GvHD and progressive disease status before transplant were significantly associated with reduced OS [68].

**Table 2:** Outcomes of additional agents added to CHOP as frontline therapy.

Author	Agent	No. of patients PTCL/AITL	ORR/CR %	PFS %	OS %
Kim SJ et al. [58]	Bortezomib	46/8	76/65	47@3years	35@3years
Ellin et al. [43] Schmitz et al. [45] d’Amore et al. [66]	Etoposide	107/18 320/28 160/30	81/NA NA 82/52.6	40@5 years. 60.7@ 3 years AITL 49@5 years	47@5 years 67.5@ 3 years AITL 52@5 years
Horwitz et al. [50]	Brentuximab vedotin	226/30	83/68	57.1@ 3 years	76.8@3years
Altmann et al. [56] Gallamini et al. [52] Kim et al. [51] Binder et al. [54] Buckstein et al. [57] Wulf et al. [55] Kluin-Nelemans et al. [53]	Alemtuzumab	127/42 24/NA 20/NA 29/NA 20/7 58/24 20/6	NA/56 75/71 80/65 60.4/58.5 68/37 72/60 90/65	33@3years 48@2 years 43.3@1year 42.4@3 years 47.5@2 years 28@3 years 27@2 years	46@3years 53@2years 44.3@1year 75.1@3 years 78.9@2 years 37@3 years 55@ 2 years
Delfau-Larue et al. [36]	Rituximab	NA/25	80/44	42@ 2 years	62@ 2 years
Ganjoo et al. [62]	Bevacizumab	39/17	90/49 reaching 53 % in AITL	44 @ 1 year 57 in AITL	88%@ 1 year in AITL
Bachy et al. [63]	Romidespin	421/NA	63/41	Median 12 months	Median 51.8 months
Zhang et al. [64]	Chidamide	113/41	PTCL 60.2/40.6 AITL 65.9/41.5	PTCL 52.4@ 3years AITL 30.3 months	PTCL 32.8@ 3years AITL 9.6 months

Abbreviations: AITL, angioimmunoblastic T cell lymphoma; PTCL, peripheral T cell lymphoma; PFS, progression free survival; OS, overall survival; ORR, overall response rate; CR, complete remission; @, at; NA, not available.

### 6.2. Relapse or Refractory AITL Therapy

Salvage chemotherapeutic protocols followed by ASCT are the standard of care in the relapsed/refractory (R/R) setting for lymphomas including PTCL, but results in PTCL patients are disappointing with relapse rates greater than 80% [69,70]. A retrospective study of 33 patients with transplant ineligible R/R PTCLs treated with ESHAP (etoposide, methylprednisolone, high-dose Ara-C, and cisplatin) as the first salvage regimen showed ORR of 46% (with CR of 39%). The median second PFS and OS were 8.0 and 11.0 months, respectively [71].

More promising outcomes are observed with alloSCT, which is associated with a 2- to 5-year PFS of 31% to 64% for nodal PTCLs [72,73,74,75,76,77,78]. Table 3 summarizes outcomes of alloSCT in AITL.

The largest data come from a recent CIBMTR retrospective analysis of 249 AITL patients who received alloSCT showed that durable remissions can be achieved even in patients who relapsed after prior ASCT. The relapse/progression, PFS, and OS rates at 4 years were 21%, 49%, and 56% respectively. Factors that were associated with the worst outcomes after allo-SCT in this study were chemoresistance status and poor performance status [79].

In 13 R/R AITL patients, BV as a single agent offered an ORR of 54% with a median PFS of 6.7 months. Interestingly, no correlation between CD30 expression and response was observed [80].

Pralatrexate showed activity in R/R PTCL irrespective of age, histologic subtypes, prior therapy and prior transplant status [81]. Based on these data pralatrexate was the first drug approved by the US Food and Drug Administration for this disease

Targeting epigenetic mutations in the R/R setting showed good disease control with better outcomes. Romidepsin is approved for the treatment of all subtypes of R/R PTCL who have received at least one prior therapy. The ORR for patients with AITL treated with romidepsin was 33%; a complete and durable response was achieved in a significant number of the patients [82]. A novel histone deacetylase inhibitor, Belinostat, had some activity in R/R PTCL patients in a phase II trial. The ORR in 120 patients was 25.8%, CR was 10.8%. Median PFS and OS were 1.6 and 7.9 months, respectively. Accordingly, the US Food and Drug Administration approved belinostat for R/R PTCL [83].

The hypomethylating agent, 5-Azacitadine, was attempted and showed sustainable responses in small AITL patient series [84,85]. Recently, in a multicenter phase II study oral azacitidine combined with romidepsin in 25 patients with R/R PTCL achieved an ORR of 61% and CR of 48% in a heavily pretreated population. Impressively, patients with T-follicular helper cell (TFH) phenotype had higher ORR (80%) and CR (67%). Better PFS was noted significantly in the presence of mutations in DNA methylation and histone deacetylation genes [86].

Lenalidomide single agent or combined with steroids could induce complete responses in patients with refractory AITL [87,88]. As monotherapy, ORR was 31% in 26 AITL patients of phase II EXPECT trial. Cyclosporine (CSA) has been used as a therapeutic option in second- or later lines with outstanding response rates [89]. 

Bendamustine as monotherapy showed activity in R/R PTCL patients, with about 50% of ORR and 25% CR. The response was short lived (3–4 months). Interestingly, AITL patients were more responsive than PTCL-NOS patients (ORR: 45.1 *versus* 20%). It was associated with OS and PFS, 4–6 months and 3 months respectively. It could represent a possible therapeutic option for elderly patients [90]. 

Interestingly, dasatinib, a multikinase inhibitor, in a preclinical trial showed better survival through inhibition of hyperactivated TCR signaling. As a monotherapy at a dose of 100 mg/body once a day in phase I clinical study, 4 of 5 RR AITL patients achieved partial responses [91]. JAK/STAT pathway is active in T cell lymphoma. Ruxolitinib, JAK 1/2 inhibitor, showed 33% ORR in R/R 9 AILT among 53 PTCL patients; responders had markedly lower pS6 expression [92].

The checkpoint pathway seems to be overactive in PTCL and AITL [93]. Checkpoint inhibitors, as single agents, in phase 2 studies showed modest activity [94,95]. ORR was around 30%. They were associated with OS 7–10 months and PFS 1–3 months. Recently, geptanolimab (GB226), an anti-PD-1 antibody, demonstrated an ORR of 40.4% in R/R PTCL. A subgroup analysis showed better response and survival in patients with PD-L1 ≥ 50% [96]. A combination of checkpoint inhibitors with other agents could be a promising option to enhance anti-tumor activity in T cell lymphoma [97]. Pembrolizumab combined with romidepsin in phase I/II trial of 15 patients with R/R PTCL revealed ORR of 44%, durable remission noted in three patients obtained a CR [98].

**Figure 1:**
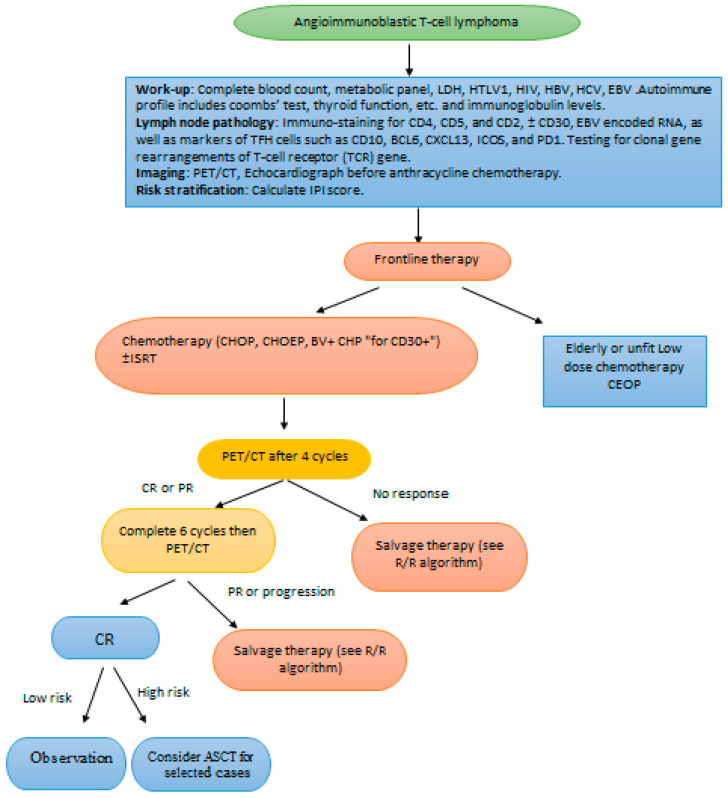
Suggested algorithm of diagnosis and frontline therapy of AITL. Notes: This algorithm is proposed to guide therapy in AITL. ASCT in CR1 is to be offered in selected patients. Abbreviations: AITL, angioimmunoblastic T cell lymphoma; PFS, progression free survival; OS, overall survival; CR, complete remission; PR, partial remission; R/R relapsed/refractory; ASCT, autologous stem cell transplantation; ISRT, involved site radiation; C cyclophosphamide; E etoposide; H anthracycline; BV brentuximab vedotin; O oncovin; P prednisone.

**Figure 2:**
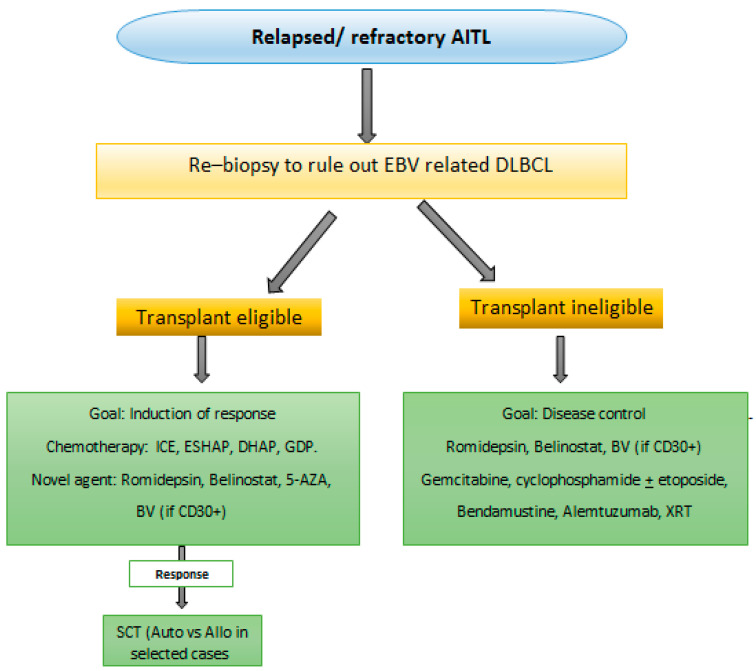
Suggested algorithm for relapsed/refractory AITL. Abbreviations: AITL, angioimmunoblastic T cell lymphoma; DLBCL, diffuse large B cell lymphoma; EBV, Epstein barr virus; Allo SCT, allogeneic stem cell transplantation; Auto SCT, autologous stem cell transplantation XRT, radiation therapy.

We propose suggested algorithms for the approach and management of AITL, Figure 1 and Figure 2. Frontline therapy includes CHOP ± E or BV + CHP. Interim PET4 is required to determine disease refractoriness. High risk AITL based on IPI are considered for ASCT in CR1. In the R/R setting re-biopsy is mandatory to rule out EBV-related BCL development. Monotherapies are not satisfactory; combinations including novel agents are encouraged. AlloSCT is suggested for prolonged survival for transplant eligible patients.

## 7. Future Directions and Ongoing Trials

A better understanding of the biologic pathways implicated in the pathogenesis of AITL and other major PTCL subtypes has encouraged many clinical trials including novel targeting therapies in both frontline and the R/R setting, listed in Table 4. 

Phase 2 trials of BV are ongoing, in the frontline with rituximab as a chemotherapy free regimen for CD30+ and/or EBV+ Lymphomas [NCT01805037], as consolidation after induction with CHEP-BV in patients with CD30-positive PTCL [NCT03113500] and as a single agent in R/R CD30 Low (<10%) mature T cell lymphoma [NCT02588651].

Epigenetic modulators are included in several trials, such as romidepsin as the first line; with CHOEP before SCT for young patients [NCT02223208], with lenalidomide [NCT02232516]. Chidamide is studied as the first line with CHOP [NCT03853044], with intravenous azacitidine for unfit patients [NCT04480125], as part of regimen in R/R with CHOEP [NCT03617432], with lenalidomide [NCT04319601]. Oral azacitidine in R/R patients as monotherapy [NCT03703375] versus romidepsin or gemcitabine is currently being investigated. [NCT01261247] is assessing panobinostat, HDAC inhibitor, in R/R PTCL patients. 

Targeting T cell signaling pathways has been investigated for PTCL; PI3K inhibitors, such as duvelisib in R/R setting as monotherapy [NCT03372057], in combination with romidepsin or bortezomib [NCT02783625], with ruxolitinib [NCT05010005]. In the first line therapy, added to CHO(E)P [NCT04803201]. [NCT02520791] is evaluating MEDI−570, an anti-ICOS monoclonal antibody in ICOS-PI3K pathways, for the follicular variant of PTCL-NOS and AITL. 

Other targeted agents tested in R/R PTCL include BCL2 inhibitors (e.g., venetoclax as single agent [NCT03552692]); JAK1/2 inhibitor ruxolitinib [NCT02974647], IDH2 inhibitors as enasidenib [NCT02273739]; monoclonal antibodies targeting CD38 (e.g., daratumumab combined with GDP[NCT04251065]); rituximab combined with lenalidomide and chidamide for R/R AITL [NCT04319601] and checkpoint inhibitors for immune modulation (e.g., pembrolizumab with pralatrexate [NCT03598998], with romidepsin [NCT03278782]).

CAR-T therapy is approved in the treatment of B cell malignancies; identifying targets for treating T cell lymphomas with such a strategy is challenging. TRBC1 is positive in 36.4% AITL patients [99]. [NCT03590574] is evaluating the safety and clinical activity of AUTO4, a CAR T cell Treatment Targeting TRBC1, in Patients with R/R TRBC1 positive selected TCL, as well, anti CD30 CAR-T [NCT04008394].

## 8. Conclusions

Options for frontline therapy and in the R/R setting are still unsatisfactory. Upfront SCT after disease is improving survival; prospective trials are warranted to confirm such benefits. A better understanding of evolving genomics for AITL allows introducing novel targeted therapies with promising outcomes. Novel assessment models including disease’s immune-histochemical features and genomics in addition to patients’ factors are needed for well-tailored treatment decisions.

## Figures and Tables

**Table 3:** Outcomes of Allogeneic stem cell transplantation in AITL.

Author	No. of Patients PTCL/AITL	OS %	PFS %
Smith et al. [72]	126/12	83@3 years	67@3years
Le Gouill et al. [73]	77/11	57(80)@5years **	53(80)@5years **
Mehta-shah et al. [75]	65/11	59@2years	48@2years
Jacobsen et al. [74]	52/5	41@3years	30@3years
Mamez et al. [68] *	285/83	67@2years	64@2years
Dodero et al. [76]	52/9	66@5years	44@5years
Kyriakou et al. [77]	0/45	64@3years	54@3years
Corradini et al. [78]	17/4	81@3years	64@3years
Epperla et al. [79]	NA/249	56@4years	49@4years

* Frontline Allo-SCT; ** PTCL(AITL). Abbreviations: AITL, angioimmunoblastic T cell lymphoma; PTCL, peripheral T cell lymphoma; PFS, progression free survival; OS, overall survival; @, at; NA not available.

**Table 4:** Ongoing Clinical trials of angioimmunoblastic T cell lymphoma.

ClinicalTrials.gov Identifier	Title	Disease Status	Intervention	Status
NCT03853044	A Phase 2, open-label study to evaluate the safety and efficacy of chidamide combined with CHOP (Cyclophosphamide, Doxorubicin, Vincristine, Prednisone) in untreated subjects with AITL	First line	Chidamide + CHOP; single arm	Recruiting
NCT02879526	A phase 2, chidamide combined with cyclophosphamide, prednisone, thalidomide in treatment of fragile patients with R/R PTCL	R/R	Chidamide + CPT; single arm	Recruiting
NCT03617432	A phase 2, chidamide combined with CHOPE regimen for PTCL Patients	R/R	Chi + CHOPE; single arm	Recruiting
NCT03593018	Randomized phase 3 study evaluating the efficacy and the safety of oral azacitidine (CC-486) Compared to investigator’s choice therapy in patient with relapsed or refractory AITL	R/R	Oral Azacitidine Vs Romedpsin or Bendamustine or Gemcitabine	Recruiting
NCT01998035	A phase 1/2; romidepsin plus oral 5-Azacitidine in relapsed/refractory lymphoid malignancies	R/R	Oral azacitadine + romidepsin	Terminated (PI left institution)
NCT04480125	A phase 2, azacitidine iv combined with chidamide in the treatment of newly diagnosed PTCL unfit for conventional chemotherapy	First line	Azacitadine + Chidamide; single arm	Recruiting
NCT04251065	A phase 2, open label, multicenter trial of Daratumumab in combination with gemcitabine, dexamethasone and cisplatin (D-GDP) in patients with relapsed/refractory CD38 positive PTCL-NOS, AITL and other nodal lymphomas of TFH cell origin	R/R	D-GDP; single arm	Not yet recruiting
NCT02520791	A phase I trial of MEDI-570 in patients with relapsed/refractory PTCL follicular variant and AITL	R/R	MEDI-570 (ICOS monoclonal antibody); single arm	Recruiting
NCT04319601	A single-arm, multiple centers, phase II study evaluating Rituximab in combination with chidamide and lenalidomide for relapsed or refractory AITL	R/R	Rituximab + Chidamide + Lenalidomide; single arm	Recruiting
NCT03703375	Randomized phase 3 study evaluation the efficacy and safety of oral azacitidine(CC-486) compared to investigator’s choice therapy in patients with relapsed or refractory AITL	R/R	Oral Azacitadine vs Romedepsin or Gemcitabine	Recruiting
NCT03552692	Use of venetoclax as single agent in patients with relapsed/refractory BCL-2 Positive peripheral T cell lymphoma.	R/R	Venetoclax; single arm	Terminated
NCT03590574	A single arm, open label, multi-center, phase I/II study evaluating the safety and clinical activity of AUTO4, a CAR T cell treatment targeting TRBC1, in patients with relapsed or refractory TRBC1 positive selected T cell non-Hodgkin lymphoma	R/R	AUTO4 (CAR T cell against TRBC1); single arm	Recruiting
NCT01719835	CHEMO-T: Cyclophosphamide, Doxorubicin, Vincristine and Prednisolone (CHOP) versus Gemcitabine, Cisplatin and Methyl Prednisolone (GEM-P) in the first line treatment of T cell lymphoma, a multicenter randomized phase II study	First line	CHOP vs GEM-P	Active not Recruiting
NCT02223208	Romidepsin in combination with CHOEP as first line treatment before Hematopoietic Stem Cell Transplantation in young patients with nodal peripheral T cell lymphomas: a phase I-II study	First line	Romidepsin + CHOEP; single arm	Recruiting
NCT03598998	A phase 1/2 study of Pembrolizumab plus Pralatrexate for treatment of relapsed or refractory PTCL	R/R	Pemrbroliumab + Pralatrexate; single arm	Recruiting
NCT02588651	A phase II study of single agent Brentuximab Vedotin in relapsed/refractory CD30 Low (<10%) mature T cell lymphoma (TCL)	R/R	Brentuximb Vedotin; single arm	Recruiting
NCT04447027	A phase 1 study of Romidepsin, CC-486 (5-azacitidine), Dexamethasone, and Lenalidomide (RAdR) for relapsed/refractory T cell malignancies	R/R	RAdR; single arm	Not yet recruiting
NCT01755975	A phase1/2; Romidepsin in combination with Lenalidomide in adults with relapsed or refractory lymphomas and myeloma	R/R	Romidepsin + Lenalidomide	Active, not recruiting
NCT02783625	A phase 1; trial of Duvelisib in combination with either Romidepsin or Bortezomib in relapsed/refractory T cell lymphomas	R/R	Duvelisib + romidepsin or Bortezomib	Recruiting
NCT03372057	A multi-Center, Phase 2, open-label, parallel Cohort study of efficacy and safety of Duvelisib in Patients with relapsed or refractory PTCL	R/R	Duvelisib; single arm	Active, not recruiting
NCT04639843	A phase 1 study of Doxorubicin, CC-486 (5-azacitidine), Romidepsin, and Duvelisib (hARD) for T cell lymphoma	First line and R/R	hARD; single arm	Not yet recruiting
NCT04803201	A randomized phase II study of CHO(E)P vs CC-486-CHO(E)P vs Duvelisib-CHO(E)P in previously untreated CD30 negative peripheral T cell lymphomas	First line	CHOEP vs Duvelisib + CHOEP	Recruiting
NCT05010005	Phase I multicenter study of Ruxolitinib and Duvelisib in relapsed or refractory T- or NK-cell lymphomas	R/R	Ruxolitinib + Duvelisib	Recruiting
NCT02974647	A phase II multicenter study of Ruxolitinib in patients with T or NK cell lymphoma that has either come back or not responded to treatment	R/R	Ruxolitinib; single arm	Recruiting
NCT03017820	Phase I trial of systemic administration of Vesicular Stomatitis Virus Genetically Engineered to Express NIS and human Interferon, in Patients With relapsed or refractory multiple myeloma, acute myeloid leukemia, and T cell neoplasms	R/R	VSV-hIFNbeta-NIS; single arm	Recruiting
NCT03113500	A phase 2 study of Brentuximab Vedotin plus Cyclophosphamide, Doxorubicin, Etoposide, and Prednisone (CHEP-BV) followed by BV consolidation in patients With CD30-positive peripheral T cell lymphomas	First line	CHEP-BV followed by BV consolidation; single arm	Recruiting
NCT04008394	Efficacy and safety of anti-CD30 CAR-T therapy in patients with refractory/relapsed lymphocyte malignancies a single-center, open, single-arm clinical study.	R/R	Anti-CD30 CAR- T therapy; single arm	Recruiting
NCT02232516	Phase II study of Romidepsin Plus Lenalidomide for patients with previously untreated PTCL	First line	Romidepsin + Lenalidomide; single arm	Recruiting
NCT00416351	A Phase I/II study of Clofarabine in patients with relapsed T cell and NK-cell lymphomas	R/R	Clofarabine; single arm	Active not recruiting
NCT02168140	Phase I dose-escalation study of CPI-613, in combination with Bendamustine, in patients with relapsed or refractory T cell Non-Hodgkin Lymphoma or classic Hodgkin Lymphoma	R/R	CPI-613 + Bendamustine; single arm	Active not recruiting
NCT01261247	A phase II study of the histone deacetylase (HDAC) inhibitor LBH589 (Panobinostat) in patients with relapsed or refractory non-Hodgkin lymphoma	R/R	Panobinostat; single arm	Active not recruiting
NCT01805037	A phase I-II trial of Brentuximab Vedotin plus Rituximab as frontline therapy for patients with CD30+ and/or EBV+ lymphomas	First line	BV + R; single arm	Active not recruiting
NCT01075321	A phase I/II clinical trial of the mTor Inhibitor RAD001 (Everolimus) in combination with Lenalidomide (Revlimid) for patients with relapsed or refractory lymphoid malignancy	R/R	Everloimus + Lenalidomide; single arm	Active not recruiting
NCT01678443	A phase I study evaluating escalating doses of 90Y-BC8-DOTA (Anti-CD45) antibody followed by autologous Stem Cell Transplantation for relapsed or refractory lymphoid malignancies.	R/R	90Y-BC8-DOTA (Anti-CD45) then ASCT; single arm	Active not recruiting
NCT02561273	A phase I/II trial of CHOEP Chemotherapy plus Lenalidomide as front line therapy for patients with stage II, III and IV peripheral T cell non-Hodgkin’s lymphoma	First line	CHOEP + Lenalidomide; single arm	Active not recruiting
NCT03278782	A phase I/II study of Pembrolizumab (MK-3475) in combination with Romidepsin in patients with relapsed or refractory PTCL	R/R	Pemborolizumab + Romidepsin; single arm	Active not recruiting
NCT03493451	A Phase 2, open-label study of BGB-A317 in patients with relapsed or refractory mature T- and NK- neoplasms	R/R	BGB-A317; single arm	Active not recruiting
NCT02533700	CEOP/IVE/GDP compared with CEOP as the first-line therapy for newly diagnosed adult patients with PTCL	First line	CEOP/IVE/GDP vs CEOP	Active not recruiting
NCT04234048	A phase 1a/1b trial in relapsed/refractory T cell non-Hodgkin lymphoma to determine the safety profile, pharmacology, and maximum tolerated dose of ST-001, a Fenretinide phospholipid suspension (12.5 mg/mL) for intravenous infusion	R/R	Dose of ST-001, a Fenretinide Phospholipid; single arm, sequential assignment dose escalating	Not yet recruiting
NCT04319601	Rituximab combined With chidamide and Lenalidomide for R/R AITL	R/R	RChR; single arm	Recruiting
NCT02341014	A phase 1/2, combination therapy with Carfilzomib, Romidepsin, Lenalidomide in patients with relapsed or refractory B- and T cell lymphomas	R/R	KRoR; single arm	Active, not recruiting
NCT02273739	A phase 1/2, multicenter, open-label, dose-escalation study of AG-221 in subjects with advanced solid tumors, Including glioma, and with AITL, that harbor an IDH2 mutation	R/R	AG-221(Enasidenib); single arm	completed
